# Supplementary figures and images for: Identification and analysis of novel recessive alleles for Tan1 and Tan2 in sorghum
Source: PeerJ. 2024 May 27;12:e17438. doi: 10.7717/peerj.17438 (PMC11138519; doi:10.7717/peerj.17438)

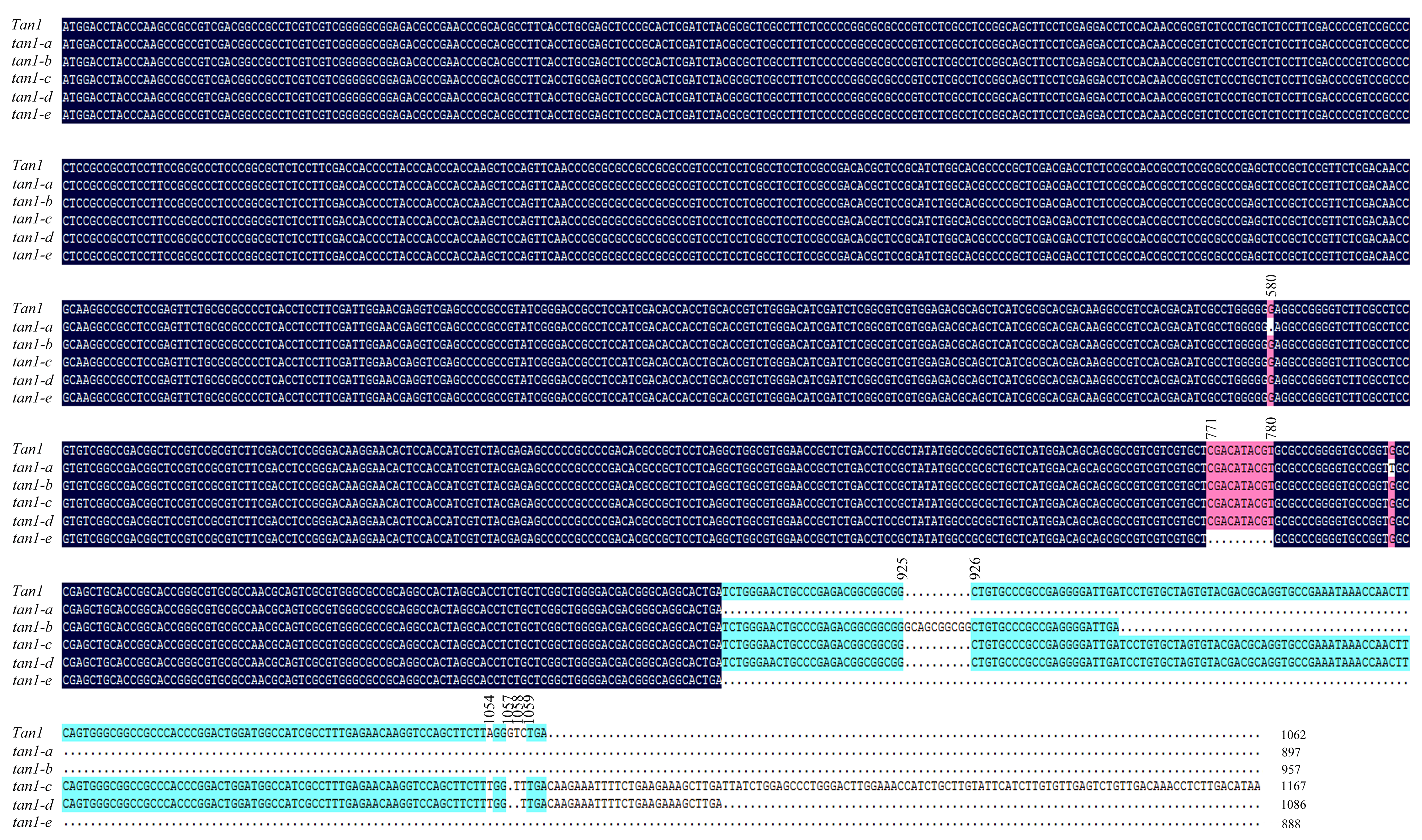

Supplement: Supplemental Information 6 — A G deletion at position 580 nt in tan1-a. 10 bp (GCAGCGGCGG) insertion between 925 and 926 nt in tan1-b. A-to-T (1,054), G deletion (1,057), and C-to-T (1,059) are changed in tan1-c. A-to-T (1,054), GT deletion (1,057 and 1,058), and C-to-T (1,059) are changed in tan1-d, then TGA (1,060, 1,061 and 1,062) stop codon alters. Sequence variation of tan1-d is similar to tan1-c. In tan1-e, 10-bp (CGACATACGT) is deleted in the coding sequence between 771 and 780. Five Tan1 alleles identified so far are shown, including tan1-a, tan1-b, and tan1-c reported previously (Wu et al., 2012, 2019) and tan1-d and tan1-e identified in this work. [file peerj-12-17438-s006.png]

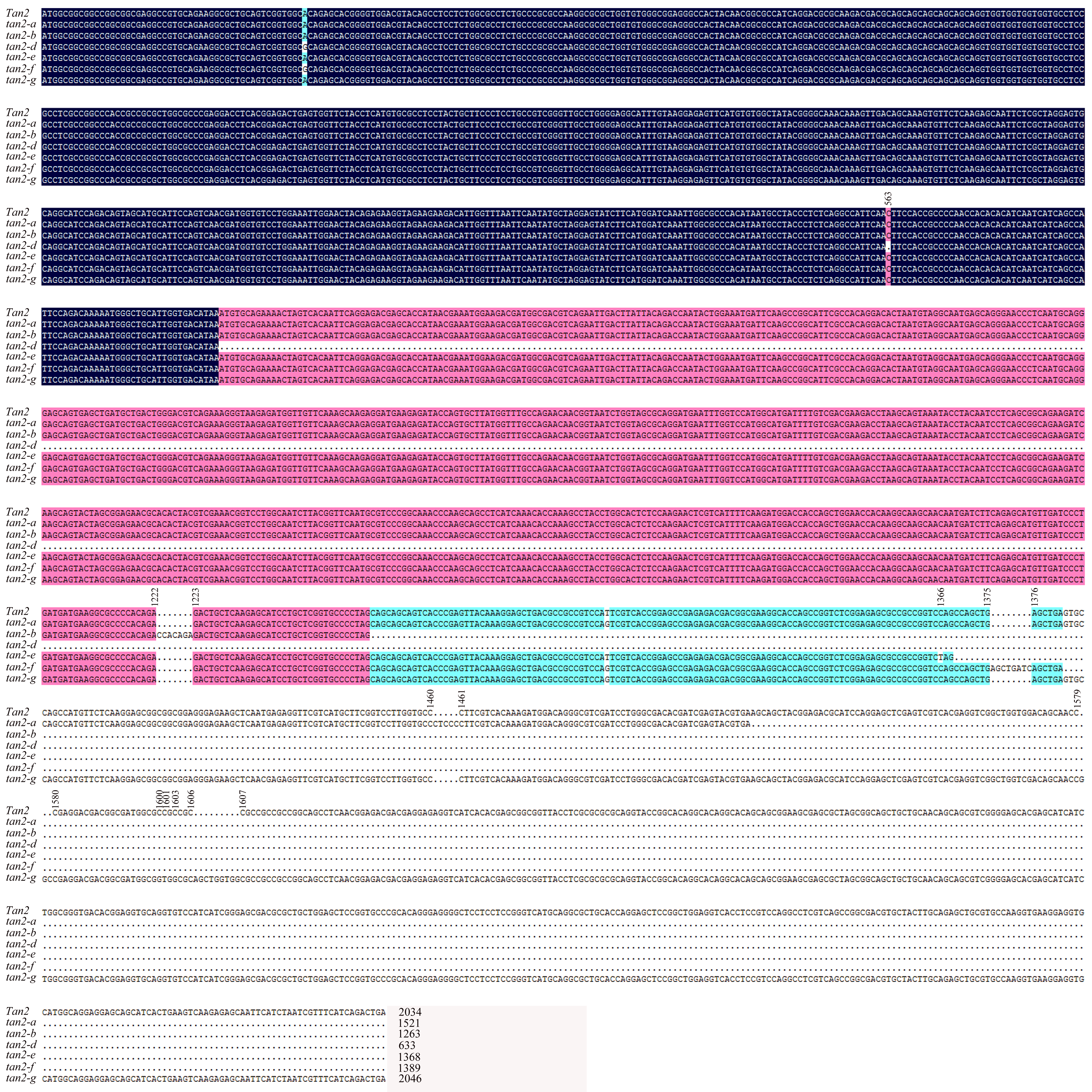

Supplement: Supplemental Information 7 — The unlined sites of difference are synonymous mutations, which don’t affect protein function. 5 bp (CTCCC) insertion between 1,460 and 1,461 nt in tan2-a. 7 bp (CCACAGA) insertion between 1,222 and 1,223 nt in tan2-b. Although 95-bp intron between exon 8 and exon 9 deletion in tan2-c, tan2-c is a recessive allele. In tan2-d, a 1-bp C deletion at position 563 in the coding region led to terminate prematurely. C-to-T transition at position 1,366 ( C AG to T AG) in the coding sequence, tan2-e results in premature termination. 8-bp (AGCTGATC) insertion between positions 1,375 and 1,376 in the coding region, tan2-f has a frameshift mutation and an early termination. tan2-g has multiple substitutions and insertions, containing 3-bp (GGC) insertion between 1,579 and 1,580, CC-to-GT at position 1,600 and 1,601, C-to-G at position 1,603 and 9-bp (AGCTGGTGG) insertion between 1,606 and 1,607 in the coding region. Seven Tan2 alleles identified so far are shown, including tan2-a, tan2-b, and tan2-c reported previously (Wu et al., 2019) and tan2-d, tan2-e, tan2-f, and tan2-g identified in this work. [file peerj-12-17438-s007.png]
