# Supplementary material for: Identification and analysis of novel recessive alleles for Tan1 and Tan2 in sorghum
Source: PeerJ. 2024 May 27;12:e17438. doi: 10.7717/peerj.17438 (PMC11138519; doi:10.7717/peerj.17438)
Supplement: Supplemental Information 9 [file peerj-12-17438-s009.docx]

| ***tan1-d* (CDS-1086 bp)**  ATGGACCTACCCAAGCCGCCGTCGACGGCCGCCTCGTCGTCGGGGGCGGAGACGCCGAACCCGCACGCCTTCACCTGCGAGCTCCCGCACTCGATCTACGCGCTCGCCTTCTCCCCCGGCGCGCCCGTCCTCGCCTCCGGCAGCTTCCTCGAGGACCTCCACAACCGCGTCTCCCTGCTCTCCTTCGACCCCGTCCGCCCCTCCGCCGCCTCCTTCCGCGCCCTCCCGGCGCTCTCCTTCGACCACCCCTACCCACCCACCAAGCTCCAGTTCAACCCGCGCGCCGCCGCGCCGTCCCTCCTCGCCTCCTCCGCCGACACGCTCCGCATCTGGCACGCCCCGCTCGACGACCTCTCCGCCACCGCCTCCGCGCCCGAGCTCCGCTCCGTTCTCGACAACCGCAAGGCCGCCTCCGAGTTCTGCGCGCCCCTCACCTCCTTCGATTGGAACGAGGTCGAGCCCCGCCGTATCGGGACCGCCTCCATCGACACCACCTGCACCGTCTGGGACATCGATCTCGGCGTCGTGGAGACGCAGCTCATCGCGCACGACAAGGCCGTCCACGACATCGCCTGGGGGGAGGCCGGGGTCTTCGCCTCCGTGTCGGCCGACGGCTCCGTCCGCGTCTTCGACCTCCGGGACAAGGAACACTCCACCATCGTCTACGAGAGCCCCCGCCCCGACACGCCGCTCCTCAGGCTGGCGTGGAACCGCTCTGACCTCCGCTATATGGCCGCGCTGCTCATGGACAGCAGCGCCGTCGTCGTGCTCGACATACGTGCGCCCGGGGTGCCGGTGGCCGAGCTGCACCGGCACCGGGCGTGCGCCAACGCAGTCGCGTGGGCGCCGCAGGCCACTAGGCACCTCTGCTCGGCTGGGGACGACGGGCAGGCACTGATCTGGGAACTGCCCGAGACGGCGGCGGCTGTGCCCGCCGAGGGGATTGATCCTGTGCTAGTGTACGACGCAGGTGCCGAAATAAACCAACTTCAGTGGGCGGCCGCCCACCCGGACTGGATGGCCATCGCCTTTGAGAACAAGGTCCAGCTTCTTTGGTTGACAAGAAATTTTCTGAAGAAAGCTTGA  **tan1-d (protein-361 aa)**  1 MDLPKPPSTA ASSSGAETPN  21 PHAFTCELPH SIYALAFSPG  41 APVLASGSFL EDLHNRVSLL  61 SFDPVRPSAA SFRALPALSF  81 DHPYPPTKLQ FNPRAAAPSL  101 LASSADTLRI WHAPLDDLSA  121 TASAPELRSV LDNRKAASEF  141 CAPLTSFDWN EVEPRRIGTA  161 SIDTTCTVWD IDLGVVETQL  181 IAHDKAVHDI AWGEAGVFAS  201 VSADGSVRVF DLRDKEHSTI  221 VYESPRPDTP LLRLAWNRSD  241 LRYMAALLMD SSAVVVLDIR  261 APGVPVAELH RHRACANAVA  281 WAPQATRHLC SAGDDGQALI  301 WELPETAAAV PAEGIDPVLV  321 YDAGAEINQL QWAAAHPDWM  341 AIAFENKVQL LWLTRNFLKK  361 A* |
| --- |
| ***tan1-e* (CDS-888 bp)**  ATGGACCTACCCAAGCCGCCGTCGACGGCCGCCTCGTCGTCGGGGGCGGAGACGCCGAACCCGCACGCCTTCACCTGCGAGCTCCCGCACTCGATCTACGCGCTCGCCTTCTCCCCCGGCGCGCCCGTCCTCGCCTCCGGCAGCTTCCTCGAGGACCTCCACAACCGCGTCTCCCTGCTCTCCTTCGACCCCGTCCGCCCCTCCGCCGCCTCCTTCCGCGCCCTCCCGGCGCTCTCCTTCGACCACCCCTACCCACCCACCAAGCTCCAGTTCAACCCGCGCGCCGCCGCGCCGTCCCTCCTCGCCTCCTCCGCCGACACGCTCCGCATCTGGCACGCCCCGCTCGACGACCTCTCCGCCACCGCCTCCGCGCCCGAGCTCCGCTCCGTTCTCGACAACCGCAAGGCCGCCTCCGAGTTCTGCGCGCCCCTCACCTCCTTCGATTGGAACGAGGTCGAGCCCCGCCGTATCGGGACCGCCTCCATCGACACCACCTGCACCGTCTGGGACATCGATCTCGGCGTCGTGGAGACGCAGCTCATCGCGCACGACAAGGCCGTCCACGACATCGCCTGGGGGGAGGCCGGGGTCTTCGCCTCCGTGTCGGCCGACGGCTCCGTCCGCGTCTTCGACCTCCGGGACAAGGAACACTCCACCATCGTCTACGAGAGCCCCCGCCCCGACACGCCGCTCCTCAGGCTGGCGTGGAACCGCTCTGACCTCCGCTATATGGCCGCGCTGCTCATGGACAGCAGCGCCGTCGTCGTGCTGCGCCCGGGGTGCCGGTGGCCGAGCTGCACCGGCACCGGGCGTGCGCCAACGCAGTCGCGTGGGCGCCGCAGGCCACTAGGCACCTCTGCTCGGCTGGGGACGACGGGCAGGCACTGA  **tan1-e (protein-295 aa)**  1 MDLPKPPSTA ASSSGAETPN  21 PHAFTCELPH SIYALAFSPG  41 APVLASGSFL EDLHNRVSLL  61 SFDPVRPSAA SFRALPALSF  81 DHPYPPTKLQ FNPRAAAPSL  101 LASSADTLRI WHAPLDDLSA  121 TASAPELRSV LDNRKAASEF  141 CAPLTSFDWN EVEPRRIGTA  161 SIDTTCTVWD IDLGVVETQL  181 IAHDKAVHDI AWGEAGVFAS  201 VSADGSVRVF DLRDKEHSTI  221 VYESPRPDTP LLRLAWNRSD  241 LRYMAALLMD SSAVVVLRPG  261 CRWPSCTGTG RAPTQSRGRR  281 RPLGTSARLG TTGRH* |
| ***tan2-d* (CDS-633 bp)**  ATGGCGGCGGCCGGCGGCGAGGCCGTGCAGAAGGCGCTGCAGTCGGTGGCGCAGAGCACGGGGTGGACGTACAGCCTCCTCTGGCGCCTCTGCCCGCGCCAAGGCGCGCTGGTGTGGGCGGAGGGCCACTACAACGGCGCCATCAGGACGCGCAAGACGACGCAGCAGCAGCAGCAGCAGGTGGTGGTGGTGGTGCCTCCGCCTCGCCGGCCCACCGCCGCGCTGGCGCCCGAGGACCTCACGGAGACTGAGTGGTTCTACCTCATGTGCGCCTCCTACTGCTTCCCTCCTGCCGTCGGGTTGCCTGGGGAGGCATTTGTAAGGAGAGTTCATGTGTGGCTATACGGGGCAAACAAAGTTGACAGCAAAGTGTTCTCAAGAGCAATTCTCGCTAGGAGTGCAGGCATCCAGACAGTAGCATGCATTCCAGTCAACGATGGTGTCCTGGAAATTGGAACTACAGAGAAGGTAGAAGAAGACATTGGTTTAATTCAATATGCTAGGAGTATCTTCATGGATCAAATTGGCGCCCACATAATGCCTACCCTCTCAGGCCATTCAATTCCACCGCCCCAACCACACACATCAATCATCAGCCATTCCAGACAAAAATGGGCTGCATTGGTGACATAA  **tan2-d (protein-210 aa)**  1 MAAAGGEAVQ KALQSVAQST  21 GWTYSLLWRL CPRQGALVWA  41 EGHYNGAIRT RKTTQQQQQQ  61 VVVVVPPPRR PTAALAPEDL  81 TETEWFYLMC ASYCFPPAVG  101 LPGEAFVRRV HVWLYGANKV  121 DSKVFSRAIL ARSAGIQTVA  141 CIPVNDGVLE IGTTEKVEED  161 IGLIQYARSI FMDQIGAHIM  181 PTLSGHSIPP PQPHTSIISH  201 SRQKWAALVT |
| ***tan2-e* (CDS-1368 bp)**  ATGGCGGCGGCCGGCGGCGAGGCCGTGCAGAAGGCGCTGCAGTCGGTGGCACAGAGCACGGGGTGGACGTACAGCCTCCTCTGGCGCCTCTGCCCGCGCCAAGGCGCGCTGGTGTGGGCGGAGGGCCACTACAACGGCGCCATCAGGACGCGCAAGACGACGCAGCAGCAGCAGCAGCAGGTGGTGGTGGTGGTGCCTCCGCCTCGCCGGCCCACCGCCGCGCTGGCGCCCGAGGACCTCACGGAGACTGAGTGGTTCTACCTCATGTGCGCCTCCTACTGCTTCCCTCCTGCCGTCGGGTTGCCTGGGGAGGCATTTGTAAGGAGAGTTCATGTGTGGCTATACGGGGCAAACAAAGTTGACAGCAAAGTGTTCTCAAGAGCAATTCTCGCTAGGAGTGCAGGCATCCAGACAGTAGCATGCATTCCAGTCAACGATGGTGTCCTGGAAATTGGAACTACAGAGAAGGTAGAAGAAGACATTGGTTTAATTCAATATGCTAGGAGTATCTTCATGGATCAAATTGGCGCCCACATAATGCCTACCCTCTCAGGCCATTCAACTTCCACCGCCCCAACCACACACATCAATCATCAGCCATTCCAGACAAAAATGGGCTGCATTGGTGACATAAATGTGCAGAAAACTAGTCACAATTCAGGAGACGAGCACCATAACGAAATGGAAGACGATGGCGACGTCAGAATTGACTTATTACAGACCAATACTGGAAATGATTCAAGCCGGCATTCGCCACAGGACACTAATGTAGGCAATGAGCAGGGAACCCTCAATGCAGGGAGCAGTGAGCTGATGCTGACTGGGACGTCAGAAAGGGTAAGAGATGGTTGTTCAAAGCAAGAGGATGAAGAGATACCAGTGCTTATGGTTTGCCAGAACAACGGTAATCTGGTAGCGCAGGATGAATTTGGTCCATGGCATGATTTTGTCGACGAAGACCTAAGCAGTAAATACCTACAATCCTCAGCGGCAGAAGATCAAGCAGTACTAGCGGAGAACGCACACTACGTCGAAACGGTCCTGGCAATCTTACGGTTCAATGCGTCCCGGCAAACCCAAGCAGCCTCATCAAACACCAAAGCCTACCTGGCACTCTCCAAGAACTCGTCATTTTCAAGATGGACCACCAGCTGGAACCACAAGGCAAGCAACAATGATCTTCAGAGCATGTTGATCCCTGATGATGAAGGCGCCCCACAGAGACTGCTCAAGAGCATCCTGCTCGGTGCCCCTAGCAGCAGCAGTCACCCGAGTTACAAAGGAGCTGACGCCGCCGTCCATTCGTCACCGGAGCCGAGAGACGACGGCGAAGGCACCAGCCGGTCTCGGAGAGCGCCGCCGGTCTAG  **tan2-e (protein-455 aa)**  1 MAAAGGEAVQ KALQSVAQST  21 GWTYSLLWRL CPRQGALVWA  41 EGHYNGAIRT RKTTQQQQQQ  61 VVVVVPPPRR PTAALAPEDL  81 TETEWFYLMC ASYCFPPAVG  101 LPGEAFVRRV HVWLYGANKV  121 DSKVFSRAIL ARSAGIQTVA  141 CIPVNDGVLE IGTTEKVEED  161 IGLIQYARSI FMDQIGAHIM  181 PTLSGHSTST APTTHINHQP  201 FQTKMGCIGD INVQKTSHNS  221 GDEHHNEMED DGDVRIDLLQ  241 TNTGNDSSRH SPQDTNVGNE  261 QGTLNAGSSE LMLTGTSERV  281 RDGCSKQEDE EIPVLMVCQN  301 NGNLVAQDEF GPWHDFVDED  321 LSSKYLQSSA AEDQAVLAEN  341 AHYVETVLAI LRFNASRQTQ  361 AASSNTKAYL ALSKNSSFSR  381 WTTSWNHKAS NNDLQSMLIP  401 DDEGAPQRLL KSILLGAPSS  421 SSHPSYKGAD AAVHSSPEPR  441 DDGEGTSRSR RAPPV |
| ***tan2-f* (CDS-1389 bp)**  ATGGCGGCGGCCGGCGGCGAGGCCGTGCAGAAGGCGCTGCAGTCGGTGGCGCAGAGCACGGGGTGGACGTACAGCCTCCTCTGGCGCCTCTGCCCGCGCCAAGGCGCGCTGGTGTGGGCGGAGGGCCACTACAACGGCGCCATCAGGACGCGCAAGACGACGCAGCAGCAGCAGCAGCAGGTGGTGGTGGTGGTGCCTCCGCCTCGCCGGCCCACCGCCGCGCTGGCGCCCGAGGACCTCACGGAGACTGAGTGGTTCTACCTCATGTGCGCCTCCTACTGCTTCCCTCCTGCCGTCGGGTTGCCTGGGGAGGCATTTGTAAGGAGAGTTCATGTGTGGCTATACGGGGCAAACAAAGTTGACAGCAAAGTGTTCTCAAGAGCAATTCTCGCTAGGAGTGCAGGCATCCAGACAGTAGCATGCATTCCAGTCAACGATGGTGTCCTGGAAATTGGAACTACAGAGAAGGTAGAAGAAGACATTGGTTTAATTCAATATGCTAGGAGTATCTTCATGGATCAAATTGGCGCCCACATAATGCCTACCCTCTCAGGCCATTCAACTTCCACCGCCCCAACCACACACATCAATCATCAGCCATTCCAGACAAAAATGGGCTGCATTGGTGACATAAATGTGCAGAAAACTAGTCACAATTCAGGAGACGAGCACCATAACGAAATGGAAGACGATGGCGACGTCAGAATTGACTTATTACAGACCAATACTGGAAATGATTCAAGCCGGCATTCGCCACAGGACACTAATGTAGGCAATGAGCAGGGAACCCTCAATGCAGGGAGCAGTGAGCTGATGCTGACTGGGACGTCAGAAAGGGTAAGAGATGGTTGTTCAAAGCAAGAGGATGAAGAGATACCAGTGCTTATGGTTTGCCAGAACAACGGTAATCTGGTAGCGCAGGATGAATTTGGTCCATGGCATGATTTTGTCGACGAAGACCTAAGCAGTAAATACCTACAATCCTCAGCGGCAGAAGATCAAGCAGTACTAGCGGAGAACGCACACTACGTCGAAACGGTCCTGGCAATCTTACGGTTCAATGCGTCCCGGCAAACCCAAGCAGCCTCATCAAACACCAAAGCCTACCTGGCACTCTCCAAGAACTCGTCATTTTCAAGATGGACCACCAGCTGGAACCACAAGGCAAGCAACAATGATCTTCAGAGCATGTTGATCCCTGATGATGAAGGCGCCCCACAGAGACTGCTCAAGAGCATCCTGCTCGGTGCCCCTAGCAGCAGCAGTCACCCGAGTTACAAAGGAGCTGACGCCGCCGTCCAGTCGTCACCGGAGCCGAGAGACGACGGCGAAGGCACCAGCCGGTCTCGGAGAGCGCCGCCGGTCCAGCCAGCTGAGCTGATCAGCTGA  **tan2-f (protein-462 aa)**  1 MAAAGGEAVQ KALQSVAQST  21 GWTYSLLWRL CPRQGALVWA  41 EGHYNGAIRT RKTTQQQQQQ  61 VVVVVPPPRR PTAALAPEDL  81 TETEWFYLMC ASYCFPPAVG  101 LPGEAFVRRV HVWLYGANKV  121 DSKVFSRAIL ARSAGIQTVA  141 CIPVNDGVLE IGTTEKVEED  161 IGLIQYARSI FMDQIGAHIM  181 PTLSGHSTST APTTHINHQP  201 FQTKMGCIGD INVQKTSHNS  221 GDEHHNEMED DGDVRIDLLQ  241 TNTGNDSSRH SPQDTNVGNE  261 QGTLNAGSSE LMLTGTSERV  281 RDGCSKQEDE EIPVLMVCQN  301 NGNLVAQDEF GPWHDFVDED  321 LSSKYLQSSA AEDQAVLAEN  341 AHYVETVLAI LRFNASRQTQ  361 AASSNTKAYL ALSKNSSFSR  381 WTTSWNHKAS NNDLQSMLIP  401 DDEGAPQRLL KSILLGAPSS  421 SSHPSYKGAD AAVQSSPEPR  441 DDGEGTSRSR RAPPVQPAEL  461 IS* |
| ***tan2-g* (CDS-2046 bp)**  ATGGCGGCGGCCGGCGGCGAGGCCGTGCAGAAGGCGCTGCAGTCGGTGGCACAGAGCACGGGGTGGACGTACAGCCTCCTCTGGCGCCTCTGCCCGCGCCAAGGCGCGCTGGTGTGGGCGGAGGGCCACTACAACGGCGCCATCAGGACGCGCAAGACGACGCAGCAGCAGCAGCAGCAGGTGGTGGTGGTGGTGCCTCCGCCTCGCCGGCCCACCGCCGCGCTGGCGCCCGAGGACCTCACGGAGACTGAGTGGTTCTACCTCATGTGCGCCTCCTACTGCTTCCCTCCTGCCGTCGGGTTGCCTGGGGAGGCATTTGTAAGGAGAGTTCATGTGTGGCTATACGGGGCAAACAAAGTTGACAGCAAAGTGTTCTCAAGAGCAATTCTCGCTAGGAGTGCAGGCATCCAGACAGTAGCATGCATTCCAGTCAACGATGGTGTCCTGGAAATTGGAACTACAGAGAAGGTAGAAGAAGACATTGGTTTAATTCAATATGCTAGGAGTATCTTCATGGATCAAATTGGCGCCCACATAATGCCTACCCTCTCAGGCCATTCAACTTCCACCGCCCCAACCACACACATCAATCATCAGCCATTCCAGACAAAAATGGGCTGCATTGGTGACATAAATGTGCAGAAAACTAGTCACAATTCAGGAGACGAGCACCATAACGAAATGGAAGACGATGGCGACGTCAGAATTGACTTATTACAGACCAATACTGGAAATGATTCAAGCCGGCATTCGCCACAGGACACTAATGTAGGCAATGAGCAGGGAACCCTCAATGCAGGGAGCAGTGAGCTGATGCTGACTGGGACGTCAGAAAGGGTAAGAGATGGTTGTTCAAAGCAAGAGGATGAAGAGATACCAGTGCTTATGGTTTGCCAGAACAACGGTAATCTGGTAGCGCAGGATGAATTTGGTCCATGGCATGATTTTGTCGACGAAGACCTAAGCAGTAAATACCTACAATCCTCAGCGGCAGAAGATCAAGCAGTACTAGCGGAGAACGCACACTACGTCGAAACGGTCCTGGCAATCTTACGGTTCAATGCGTCCCGGCAAACCCAAGCAGCCTCATCAAACACCAAAGCCTACCTGGCACTCTCCAAGAACTCGTCATTTTCAAGATGGACCACCAGCTGGAACCACAAGGCAAGCAACAATGATCTTCAGAGCATGTTGATCCCTGATGATGAAGGCGCCCCACAGAGACTGCTCAAGAGCATCCTGCTCGGTGCCCCTAGCAGCAGCAGTCACCCGAGTTACAAAGGAGCTGACGCCGCCGTCCAGTCGTCACCGGAGCCGAGAGACGACGGCGAAGGCACCAGCCGGTCTCGGAGAGCGCCGCCGGTCCAGCCAGCTGAGCTGAGTGCCAGCCATGTTCTCAAGGAGCGGCGGCGGAGGGAGAAGCTCAACGAGAGGTTCGTCATGCTTCGGTCCTTGGTGCCCTTCGTCACAAAGATGGACAGGGCGTCGATCCTGGGCGACACGATCGAGTACGTGAAGCAGCTACGGAGACGCATCCAGGAGCTCGAGTCGTCACGAGGTCGGCTGGTCGACAGCAACCGGCCGAGGACGACGGCGATGGCGGTGGCGCAGCTGGTGGCGCCGCCGCCGGCAGCCTCAACGGAGACGACGAGGAGAGGTCATCACACGAGCGGCGGTTACCTCGCGCGCGCAGGTACCGGCACAGGCACAGGCACAGCAGCGGAAGCGAGCGCTAGCGGCAGCTGCTGCAACAGCAGCGTCGGGGAGCACGAGCATCATCTGGCGGGTGACACGGAGGTGCAGGTGTCCATCATCGGGAGCGACGCGCTGCTGGAGCTCCGGTGCCCGCACAGGGAGGGGCTCCTCCTCCGGGTCATGCAGGCGCTGCACCAGGAGCTCCGGCTGGAGGTCACCTCCGTCCAGGCCTCGTCAGCCGGCGACGTGCTACTTGCAGAGCTGCGTGCCAAGGTGAAGGAGGTGCATGGCAGGAGGAGCAGCATCACTGAAGTCAAGAGAGCAATTCATCTAATCGTTTCATCAGACTGA  **tan2-g (protein-681 aa)**  1 MAAAGGEAVQ KALQSVAQST  21 GWTYSLLWRL CPRQGALVWA  41 EGHYNGAIRT RKTTQQQQQQ  61 VVVVVPPPRR PTAALAPEDL  81 TETEWFYLMC ASYCFPPAVG  101 LPGEAFVRRV HVWLYGANKV  121 DSKVFSRAIL ARSAGIQTVA  141 CIPVNDGVLE IGTTEKVEED  161 IGLIQYARSI FMDQIGAHIM  181 PTLSGHSTST APTTHINHQP  201 FQTKMGCIGD INVQKTSHNS  221 GDEHHNEMED DGDVRIDLLQ  241 TNTGNDSSRH SPQDTNVGNE  261 QGTLNAGSSE LMLTGTSERV  281 RDGCSKQEDE EIPVLMVCQN  301 NGNLVAQDEF GPWHDFVDED  321 LSSKYLQSSA AEDQAVLAEN  341 AHYVETVLAI LRFNASRQTQ  361 AASSNTKAYL ALSKNSSFSR  381 WTTSWNHKAS NNDLQSMLIP  401 DDEGAPQRLL KSILLGAPSS  421 SSHPSYKGAD AAVQSSPEPR  441 DDGEGTSRSR RAPPVQPAEL  461 SASHVLKERR RREKLNERFV  481 MLRSLVPFVT KMDRASILGD  501 TIEYVKQLRR RIQELESSRG  521 RLVDSNRPRT TAMAVAQLVA  541 PPPAASTETT RRGHHTSGGY  561 LARAGTGTGT GTAAEASASG  581 SCCNSSVGEH EHHLAGDTEV  601 QVSIIGSDAL LELRCPHREG  621 LLLRVMQALH QELRLEVTSV  641 QASSAGDVLL AELRAKVKEV  661 HGRRSSITEV KRAIHLIVSS  681 D* |
